# Supplementary material for: Identification and functional analysis of non-coding regulatory small RNA FenSr3 in Bacillus amyloliquefaciens LPB-18
Source: PeerJ. 2023 May 15;11:e15236. doi: 10.7717/peerj.15236 (PMC10194069; doi:10.7717/peerj.15236)
Supplement: Supplemental Information 4 [file peerj-11-15236-s004.zip › KO/CK-vs-T1_map/map04139.html]

KEGG PATHWAY: Mitophagy - yeast - Reference pathway


|  |  |
| --- | --- |
| **Mitophagy - yeast - Reference pathway** |  |

[
Pathway menu
| Organism menu
| Pathway entry
| Show description
| User data mapping
]

Mitophagy, which refers to the selective elimination of impaired or excessive mitochondria, is considered to be the main mechanism for mitochondria quality and quantity control. In yeast, oxidative stress or inhibition of TOR induces expression of Agt32, the indispensable mitochondrial outer membrane receptor. Direct phosphorylation of Atg32 by CK2 triggers mitophagy, and two mitogen-activated protein kinase (MAPK) signal transduction pathways are also important in this process. Atg11 is an adaptor protein for selective autophagy, and recruits the cargo to the phagophore assembly site (PAS), where the autophagosome is generated. Then Atg32-Atg8 interaction enhances the formation of the autophagosome surrounding the mitochondria, which finally fuses with vacuoles for degradation. Atg32-Atg11 interaction may also be regulated by Yme1-mediated processing of Atg32, as well as by mitochondrial fission machinery. Some factors have been suggested to positively or negatively regulate the mitophagy via different mechanisms, including Atg1, Atg33, Mss4, Fmc1, Mdm38, Mip1, and Ubp3-Bre5 deubiquitination complex.

|  |  |  |
| --- | --- | --- |
| Reference pathway | 184% 150% 122% 100% 82% 67% 55% | 图片下载 |
